# The feasibility of combining greening schoolyards and nutrition education in primary schools: A qualitative study

**DOI:** 10.1371/journal.pone.0313773

**Published:** 2024-11-15

**Authors:** Geertje van Wijk, Lisa van Antwerpen, Femke A. Hoefnagels, Sjef J. J. M. Staps, Marieke C. E. Battjes-Fries

**Affiliations:** 1 Louis Bolk Instituut, Department of Nutrition and Health, Bunnik, The Netherlands; 2 JOGG, Den Haag, The Netherlands; Sefako Makgatho Health Sciences University, SOUTH AFRICA

## Abstract

**Objective:**

An increasing number of children in the Netherlands is overweight or obese, which is largely attributable to an unhealthy lifestyle and unhealthy living environment. Nutrition education and greening the schoolyard, for example with a vegetable garden, have independently been studied and are shown to be effective in teaching children a healthy lifestyle and providing a healthy living environment. However, the feasibility of combining nutrition education and greening the schoolyard has not been studied yet. Therefore, this study aimed to provide insight into primary schools’ vision on making this combination, and the feasibility of doing so.

**Methods:**

In this study a qualitative research design was used. The theoretical frameworks of Proctor et al. and Sekhon et al. were used to develop the interview guide. Twelve semi-structured interviews were conducted with members of school teams and school directors. The interviews were transcribed verbatim and analyzed using the thematical analysis of Braun and Clarke.

**Results:**

According to the interviewees, three facets are essential to make the combination of greening schoolyards and nutrition education feasible and successful. Firstly, the interviewees mentioned that the school team and school directors of schools must be able to see the connection between greening the schoolyard and nutrition education. Additionally, support is needed among the parents, school team, pupils and local community. Finally, the interviewees stated that schools must be able to integrate greening the schoolyard and nutrition education into their existing curriculum.

**Conclusions:**

The results of this study showed that combining greening schoolyards and nutrition education in primary schools is feasible and successful when vision, support and integration are present. Future research should investigate the vision of the local community and parents on making the combination, and the effects of the combination on pupils and their environment.

## Background

The prevalence of overweight and obesity in children has been increasing for years [1]. In 2021, 15% of Dutch children aged two to twelve was overweight, and 4% was obese [2]. Overweight and obesity can lead to chronic diseases such as type 2 diabetes, as well as orthopedic problems, respiratory issues, fertility problems, cardiovascular diseases, and psychosocial consequences like negative self-image, emotional and behavioral problems, and depression [1]. Additionally, obesity at a young age predicts obesity in adulthood [3]. The increase in overweight and obesity is largely attributed to an unhealthy lifestyle, which includes unhealthy eating behavior and a decreasing knowledge about healthy food, and to an unhealthy living environment [4–6]. For instance, a lot of children nowadays eat too much fat and sugar, and their diets frequently lack sufficient amounts of fresh fruit and vegetables [6]. Additionally, the average living environment of children nowadays increasingly promotes a high energy intake and sedentary activity [7].

Research indicates that dietary habits formed early lay the foundation for eating habits and patterns in adulthood [8]. Teaching children about healthy food and a healthy eating pattern at a young age is, therefore, crucial. The primary school is a suitable place to teach children about healthy eating behavior because it provides a learning environment, almost every child attends school, children spend a significant part of their time there, and it brings together children from different social backgrounds [9, 10].

Providing nutrition education in primary schools allows children to gain nutrition skills and knowledge about the food chain and healthy eating behavior, and empowers them to make healthy food choices [11]. Another successful way to teach children a healthy eating pattern is by greening the schoolyard including establishing a vegetable garden, creating a food forest or planting berries or other edible shrubberies on the school grounds [6]. Studies have shown for example that children who participate in gardening have a greater knowledge of vegetables, eat vegetables earlier and in larger quantities, and have a more positive attitude toward vegetables than children who do not garden [12, 13]. Greening schoolyards also offers other benefits: it improves the living environment of children since they spend a considerable amount of time at school, it increases the creativity and concentration of children, children engage in more physical activity than those in a school without a green space, it enhances local biodiversity, and contributes to a better climate [14, 15]. The latter is relevant because the production and consumption of food are responsible for over 25% of total greenhouse gas emissions [16]. Adopting sustainable eating patterns can reduce this emission and also offer health benefits, such as a reduced risk of diabetes and cardiovascular diseases [16]. Given the current climate change, teaching children a healthy and sustainable eating pattern is especially crucial.

Providing nutrition education can be complemented with activities facilitated by having a vegetable garden at primary schools, also known as ‘garden-enhanced nutrition education’ or ‘garden-based nutrition education’. This enables schools in making a link between theory and practice by the use of activities such as gardening, tasting products from the garden, cooking classes, and communal meals, and therewith creating the possibility to utilize the benefits of both greening the schoolyards and nutrition education. Being able to make the connection between greening schoolyards and nutrition education, and making use of activities such as the above-mentioned, asks for a profound change in education programs in schools. Taking into account the perspectives, capabilities and wishes of the school staff in the process of greening the schoolyard and offering nutrition education therefore is essential. A limited number of studies focused on these perspectives until now, which all focus solely on greening the schoolyard or nutrition education. To the best of our knowledge, no studies in the Netherlands or internationally have yet investigated the perspectives of schools and their employees on connecting both, and the feasibility to do so. Therefore, this study aims to create insight into the views of primary school employees on combining greening schoolyards, including creating a vegetable garden, with nutrition education, and the feasibility of doing so.

## Materials and methods

### Study design

In this study, a qualitative research design was employed. To collect data, 12 semi-structured interviews were held with members of school teams and school directors. The goal of the interviews was to gain insight into schools’ perspectives on combining the greening of schoolyards and nutrition education, and the feasibility of doing so.

The interviews took place in May and June 2023. It was aimed to conduct all interviews face-to-face, however, depending on the preference of and feasibility for the interviewees, this was not always possible. Seven interviews were eventually conducted face-to-face during a visit to the school and five interviews were held remotely via Teams or phone calls. No repeat interviews were carried out. The duration of the interviews ranged from 18 to 103 minutes (with most of the interviews during approximately 40 minutes), depending on the time available of the interviewees. No notes were taken during the interviews; however, the interviews were audio-recorded after obtaining interviewee’s consent. Apart from the interviewee and the researcher (GvW), no one else was present during the interviews.

Participation in the study was voluntary. A written informed consent was obtained from the participants. The collected data was processed in a coded manner. The collection, processing, and storage of data were carried out in accordance with the GDPR implementation law. This study was exempted from METC approval by the medical ethical review committee METC-UMC.

### Participants

Convenience and purposive sampling were used. There was no pre-established relationship between the researchers and the school employees. The target number of participants was approximately twelve. The majority of participants for the interviews were recruited from schools participating in a broader effect study. The broader effect study was an implementation and effect study on combining greening schoolyards and nutrition education in primary schools, in which ten primary schools throughout the Netherlands participated. As part of that study, pupils in grade five to eight (8 to 11 year olds) participated in a questionnaire in September-October 2022 and again in June-July 2023. In addition, teachers and pupil’s parents also participated in a questionnaire in June-July 2023 (results not published). The ten schools of this broader study were recruited between the 21^st^ of April 2022 and the 31^th^ of October 2022 through recruitment texts in newsletters and via channels and networks of collaboration partners (e.g., through LinkedIn). Additionally, schools were actively approached with recruitment letters, allowing enrollment through a link or requesting additional information. Six schools ultimately enrolled in the broader effect study with plans to green their schoolyards within the schoolyear of 2022–2023. The other four schools did not have a green schoolyard nor offered nutrition education. Since the main study investigated green schoolyards and/or offer nutrition education, only the six schools from the broader effect study that had plans to green their schoolyards were asked if there were employees willing to participate in an interview for the main study. Nine employees of all six schools volunteered for an interview. Employees who declined participation cited being too busy and therefore not being able to participate.

Apart from the schools and their participants recruited via the broader effect study, three additional schools that had recently greened their schoolyards were recruited between the 1^st^ of April 2023 and the 15^th^ of May 2023 through the researchers’ own network. Similar to the previous approach, these schools were contacted to participate in an interview. Three employees of these three schools volunteered for an interview, while those who declined cited time constraints as the primary reason to decline.

To obtain a comprehensive understanding of schools’ perspectives on combining greening and nutrition education, and the feasibility of doing so, employees from participating schools at both executive (people who perform tasks related to teaching pupils, e.g., a teacher) and managerial levels (e.g., a director) were invited to participate in an interview. Employees who were involved in the school throughout the school year (2022/2023) and were willing to voluntarily participate in an interview were included. During the recruitment, variation in school characteristics such as denomination, location and size were considered to achieve a balanced representation. This was done through careful consideration of the additional schools that were recruited, taking into account the school characteristics of the six schools that were already recruited via the broader effect study.

### Outcome measures and procedures

For conducting the semi-structured interviews, an interview guide was developed (see S1 File). The topics in the interview guide were based on the theoretical frameworks of Proctor et al. [17] and Sekhon et al. [18], and on additional literature [19, 20].

Both theoretical frameworks are commonly used in research on process evaluations and implementation research to assess the feasibility and acceptance of interventions [21–24]. The framework of Proctor et al., shown in Table 1, focuses on the broader context of the implementation and evaluation of an intervention, while the framework of Sekhon et al., shown in Table 2, aims to explore the acceptance of the intervention and the personal perspectives of stakeholders on the intervention [17, 18]. By combining these frameworks, a comprehensive overview of both the broader context of implementation as well as more specific factors influencing the acceptance of, and vision on combining the greening of schoolyards with nutrition education could be obtained.

**Table 1:** Concepts from the Proctor et al. framework [17].

Concept	Description
Acceptability	The stakeholders’ perception of the pleasantness, appropriateness, and sufficiency of the implementation of a particular intervention.
Adoption	The establishment of the application of the intervention in practice.
Appropriateness	The perceived suitability, relevance, or compatibility of an intervention in a particular setting and for a specific problem.
Feasibility	The extent to which a new intervention can be successfully used or implemented within a particular setting.
Fidelity	The extent to which an intervention is carried out as intended by the initiators.
Implementation cost	The costs incurred for the implementation of the intervention.
Penetration	The portion of the target audience that uses the intervention.
Sustainability	The extent to which the implementation of the intervention is integrated and continued within the normal activities of the involved organization in which the intervention is implemented.

**Table 2:** Concepts from the Sekhon et al. framework [18].

Concept	Description
Affective attitude	Describes how stakeholders think about the intervention and what their feelings toward the intervention are.
Burden	The perceived amount of effort required to participate in or execute the intervention.
Ethicality	The extent to which the intervention aligns with the personal norms and values of the stakeholders.
Intervention coherence	The extent to which stakeholders understand the intervention and how it works.
Opportunity costs	The extent to which benefits, gains, or values must be sacrificed to participate in or carry out the intervention.
Perceived effectiveness	The extent to which the intervention is believed to achieve its goal.
Self-efficacy	The confidence of stakeholders that they can exhibit the behavior necessary to participate in or carry out the intervention.

All concepts from the Sekhon et al. framework were included in the development of the interview guide. Except for the concepts of fidelity, implementation cost, and penetration, all concepts from the Proctor et al. framework were used in the development of the interview guide. Fidelity was not included because the focus of this study was not on the extent to which the intervention was implemented as intended; schools had the freedom to decide how and what to implement. Instead, it was more relevant to understand how and why they experienced the actions they eventually took regarding greening or providing nutrition education. Implementation costs were not included because this study did not address the cost-effectiveness of implementation as it focused on the experiences and visions of schools. Penetration was not included because it was assumed that pupils had no choice about whether or not they use the intervention: in principle they all participated.

Based on these concepts and additional literature, four main topics of the interview guide were formulated: 1) vision and attitude towards nutrition education and greening the schoolyard (affective attitude, ethicality, intervention coherence, perceived effectiveness, appropriateness), 2) decision-making and implementation of nutrition education and greening (acceptability, adoption), 3) needs and barriers (burden, opportunity costs, self-efficacy), and 4) success and failure factors (feasibility, sustainability). For example, topic one included the question ‘Do you feel that greening the schoolyard and offering nutrition education has an influence on the pupils?’, topic two included ‘Are you satisfied with how your school has started with the green schoolyard and/or with offering nutrition education?’, topic three included ‘Did you encounter any barriers when using the green schoolyard, providing nutrition education or combining these?’ and the fourth topic included ‘What do you think are the success factors or tips for getting started with a greener schoolyard, working with nutrition education or both?’.

Besides these four topics, the interview guide also consisted of questions meant to gain insight in the current situation of schools, such as the current activities and policies of schools regarding greening the schoolyard and nutrition education. For instance, schools were asked if they make use of the ‘Healthy School’ approach, which is a national program that supports schools to work on specific health topics such as nutrition [25].

Before conducting the interviews, the interview guide was discussed with researchers from the project group of this study. Additionally, the interview was pilot tested with the researchers from this project group that have experience with executing qualitative research in schools, to become familiar with the interview questions and the structure of the interview. To reduce the risk of research bias, the interviewer also used open questions and follow-up questions (probing) based on the participant’s answers.

### Data analysis

All interviews were audio-recorded, anonymized and transcribed verbatim. Using the software program Atlas.ti (version 23) and Braun and Clarke’s thematic analysis [26], the transcripts were inductively and deductively coded by the main researcher (GvW) (see S2 File for the code book). The codes, predefined based on the theoretical frameworks of Proctor et al. and Sekhon et al. after being discussed by GvW with two researchers from the project group (FAH and MCEB), and supplemented with codes emerging from the data, were then categorized. The categorization of the codes was then discussed by GvW with another researcher (FAH). Based on the categorization, themes were identified and discussed by GvW with two researchers from the project group (FAH and MCEB), after which a summary was created for each theme. Recommendations and conclusions were then drawn from these summaries. By discussing every step of the analysis with researchers from the project group and therewith assuring research triangulation, as well as by consistently documenting in an audit trial, data trustworthiness and credibility was ensured.

After 12 interviews, no new information was acquired from the interviews, which was determined by comparing the codes and identified themes, indicating data saturation and therewith the adequacy of the sample [27]. The transcripts were not returned to participants. After finalization of the study, participants received a letter with the main results of the study. Subsequently, several participants provided feedback on the results of the study. No discrepancies were found between the letter and the feedback from the participants.

## Results

### Characteristics of the study sample

Twelve interviews were conducted at nine primary schools. The study population consisted of six interviewees with an executive function, such as a teacher or garden coach, and six school directors. Table 3 shows the characteristics of the participating schools and interviewees. No difference was found in the data between the responses of interviewees with an executive function and those with a managerial function. Therefore, in the results no distinctions have been made between interviewees unless indicated specifically.

**Table 3:** Characteristics of the interviewees and primary schools.

		Interviewees (n = 12)	Primary schools (n = 9)
Gender (n, %)	*Men* *Women*	2 (17%) 10 (83%)	
Form of employment (n, %)	*Executive* *Managerial*	6 (50%) 6 (50%)	
Denomination (n, %)	*Public* *Christian* *Islamic*		2 (22%) 6 (66%) 1 (11%)
School size (n, %)	*Small (≤100 pupils)* *Medium (101–199 pupils)* *Large (200≥ pupils)*		2 (22%) 3 (33%) 4 (44%)
Type of education (n, %)	*Primary education* *Special needs primary education*		8 (89%) 1 (11%)
Educational concept (n, %)	*Regular* *Other*, *e*.*g*. *Montessori*, *Vrijeschool*		4 (44%) 5 (55%)
Healthy School-certificate (n, %)	*Yes* *No*		3 (33%) 6 (66%)

Of the nine schools participating in this study, four schools had fully greened their schoolyards within the schoolyear 2022–2023 and had a vegetable garden on or around the schoolyard, three schools had partially greened, whereof one had a vegetable garden nearby, and two schools had not been able to green their schoolyard despite planning to do so. Of these last two schools, one had a vegetable garden nearby that they used.

Factors such as having to share the schoolyard with other schools, uncertainty about the school’s and the schoolyard’s future, soil pollution, or having a schoolyard that is municipal property have delayed or prevented the process of greening the schoolyard or making the connection with nutrition education for some schools in this study.

All nine schools provided some form of nutrition education, but the extent in which they did so, varied between the schools. For instance, all schools had a fruit policy or participated in the European school fruit scheme program for which they receive subsidy to offer pupils at least three days in a week free fruit during 20 weeks. Furthermore, most schools paid attention to water consumption, a few schools offered school lunches, half of the schools provided lessons on healthy and sustainable nutrition, and some schools engaged in gardening.

Three out of the nine schools already made a connection between greening the schoolyard and nutrition education. For instance, by using the vegetable garden for cooking lessons or by tasting products from the garden and linking this to theory about tastes. Furthermore, three schools intended to make this connection or strengthen this connection in the near future, and three schools did not make a connection and had no plans to establish this. Three of the nine schools had a Healthy School-certificate.

### Vision: How schools perceive the relationship between greening the schoolyard and nutrition education

The interviews reveal that schools’ views on combining greening the schoolyard and nutrition education are playing a crucial role in making it feasible to link greening the schoolyard and nutrition education. Schools that want to make or already make the connection do not see greening the schoolyard separately from nutrition education but rather as a whole or an extension of each other. When interviewees from these schools were asked why they see it this way, no clear reason was formulated: *"yes*, *that’s just one and one*,*" "that’s actually very simple*,*" "one cannot really do without the other*,*"* and *"yes*, *that kind of flows into each other for me*.*"* For these interviewees, making the connection between greening the schoolyard and nutrition education is self-evident.

Interviewees from schools that do not make and do not want to make the connection between greening the schoolyard and nutrition education indicated that they do not see a coherence between the two. Similar to the participants that do see coherence but could not explain why, these interviewees could also not explain why they do not see any coherence. Interviewees mentioned that they simply do not see the connection and do not find it self-evident that there would be a connection between greening and nutrition education, making it unfeasible to make the connection and combine greening the schoolyard and nutrition education.

*On a school level, I don’t actually see the connection. In the sense of, we do have our policy of doing it as healthy as possible, but I don’t see the connection between the vegetable garden and healthy eating*." *(Interviewee 1, school 1)*

Apart from some interviewees who stated to not see any connection, most interviewees said they do find it important and relevant that their school is involved in greening and/or nutrition education. Additionally, engaging with themes related to greening, nutrition, and health personally aligned with the values of most interviewees. Also, apart from where the interviewees were standing in the process of greening the schoolyard and/or offering nutrition education, the majority of the interviewees mentioned the same general tip for other schools: if you start with greening the schoolyard or with nutrition education, you should work in small steps and be content with every step you can take. Start small and then work towards your goal step by step.

### Support needed to make the connection between greening and nutrition education

The interviewees highlighted that support from the parents, school team, pupils, and local community is another crucial aspect that is needed to make it feasible and more successful to connect greening the schoolyard and nutrition education. According to all interviewees, the way in which support arises and has influence on the feasibility and successfulness of connecting greening and nutrition education, differs between the parents, school team, pupils, and local community.

### Parental support

Support among parents is generally high when it comes to greening the schoolyard, but lower when it comes to nutrition education. Most interviewees indicated that parents regularly help with the establishment and maintenance of the greened schoolyard, gardening with the pupils, and contributing materials for the schoolyard. Parents do this on a voluntary basis. One interviewee mentioned the following about parental support for greening the schoolyard:

*"That is only positive. Really, only positive. People who really say how beautiful it will be, how fun. And how nice, and outdoors. Yes, really, only positive. And when I announced that we wanted to create an indoor forest, and we were like, do you want to give us some plants? Well, I went crazy. I had so many plants, really. I didn’t know where to put them. But very nice*." *(Interviewee 2, school 2)*

On the other hand, most interviewees mentioned that parental support for nutrition education is lower. According to the majority of the interviewees, parents often seem to have a different perspective on what healthy eating means than the school does, and they want to decide for themselves what their child eats, according to their own understanding of healthy eating. Introducing the school’s vision on healthy eating step by step and gradually offering more nutrition education is, according to several interviewees, the best way to cope with this situation.

*"Yes, that is a hindrance in that sense. Because you enter an area of which parents think it’s their area, and I can agree that it is an area of the parents. But with those small steps, it works. If you go too far, you can see that you won’t get people along, then parents will say ’yes, you’re in my parenting area’. Yes, that’s the most difficult*." *(Interviewee 3, school 3)*

### Support by the school team

Regarding support within the school team, the interviews reveal that there is little difference between support for greening the schoolyard or for nutrition education. According to most interviewees, support within the school team is mainly influenced by barriers such as budget, manpower, and mainly time, and less by a personal vision. Several interviewees mentioned that nowadays, so much is asked of primary schools that time is the biggest obstacle in greening the schoolyard and making use of the greened schoolyard as well as in offering nutrition education, thus also hindering the feasibility of connecting both.

*"Schedules are packed. You have to do everything, and then teachers feel like I have to do this too, yes, I don’t have time. I think that is the stumbling block, a lack of time*." *(Interviewee 4, school 4)*

Furthermore, all interviews showed that support within the school team, compared to support in the other groups of people, is the most essential for successfully executing greening the schoolyard and nutrition education. According to several interviewees, greening and nutrition education should be supported by the team so that it can be institutionalized in the school’s culture. Several interviewees mentioned that if it is institutionalized in the school’s culture, it becomes clearer for both parents and the school team what they choose when they choose for that specific school, and that this increases support among parents and interviewees who consciously choose for that culture.

*"If you want to leave it only to a school team, well, then it is doomed to fail from the start*." *(Interviewee 4, school 4)*

To strengthen support within the school team, several interviewees mentioned the importance of having a leader. Additionally, specifically for greening the schoolyard some schools have a ’green committee’ or a ’green working group,’ consisting of team members and parents, and possibly pupils, to ensure involvement and therewith enlarging support. Multiple interviewees also mentioned that they believed that the government can play an important role in facilitating greening the schoolyard and offering nutrition education, since the government can change the content of the training that primary teachers need to follow before starting to work. According to the interviewees, if themes as greening and nutrition education would already be included within this training, it would make it much easier for teachers to start working on these themes, as they would gain more background knowledge about it and get normalized with teaching these themes in primary school.

### Pupils support

In addition to support among parents and the school team, support among pupils also plays a role in the feasibility and successfulness of making the connection. All interviewees indicated that there is generally strong support among pupils for working with greening and nutrition education. According to all interviewees, pupils react very enthusiastically when they can cook, receive lessons on healthy eating, or participate in gardening in school. One interviewee mentioned that especially classes of grade four and five (7 to 9 year olds) find gardening very enjoyable. This difference between grades was not apparent in other schools.

All interviewees also believed that when there is support among pupils, engaging in greening the schoolyard and nutrition education has an extra positive effect on pupils compared to when there is little support. According to the interviewees, when there is support among pupils, the subject matter is better retained, and engaging with the themes of greening, food origin, and healthy eating becomes normalized. Several interviewees also mentioned that the teacher’s enthusiasm plays a role in the pupils’ enthusiasm, and vice versa, and that the school team’s support can in this way be related to pupils’ support.

*"Look, telling about it is one thing. But if you actually implement it in practice and do something with it, that has much more impact on pupils*." *(Interviewee 5, school 5)*

To strengthen support among pupils, a pupil’s council can be established. According to several interviewees, this creates a sense of responsibility, so pupils encourage each other to participate or to be careful with the vegetable garden, for example. In addition, involving pupils more in the process can increase enthusiasm among pupils.

*"What I do notice, because I created the garden with the class, they were also a bit proud of that vegetable garden, however small it was, that they also have a bit of social control on the playground. Like, be careful there because there are plants. That’s yours, protect it well. You’ve worked hard on that*." *(Interviewee 6, school 6)*

### Support by the local community

Finally, support in the school’s local community also plays a role in de feasibility and success of combining greening the schoolyard and nutrition education, as this support can make it especially more feasible to create, have and use a green schoolyard. Most interviewees mentioned that an involved local community, such as the immediate neighbors next to the school, the neighborhood wherein the school is located, or the shops near the school, facilitates the maintenance of the greened schoolyard. Volunteers from the local community can be sought to help in the garden or water the plants during holidays, so it is not entirely dependent on parents. Additionally, several interviewees mentioned that an involved local community can also help against vandalism by keeping an eye on the schoolyard, even outside school hours.

*“Imagine it [the greenhouse] comes here, then you also need a bit of social control from the local community. I think if it becomes something for all of us, if a neighbor has an interest in it, they might provide volunteers to help. So, I try to aim for that, so that you can also combat vandalism a bit*." *(Interviewee 7, school 2)*

To involve the local community with the schoolyard, several interviewees mentioned that a sense of shared responsibility must be created. This can be done, for example, by opening the school’s vegetable garden to the local community so that people from the this community can also benefit. Additionally, one interviewee mentioned that, especially for schools in cities, it is good to make the local community aware that a green schoolyard also brings benefits to the community itself, such as reducing heat stress, creating a good reason for people from the local community to come and help.

*"I think if you show what you’re working on, that it radiates something communal. If we really radiate that to the local community, for example like hey come by our school because we’re going to make a box of herbs now, come pick herbs with us. Or we have lettuce left from the vegetable garden because you harvested too much, that way you can involve the local community a bit*." *(Interviewee 8, school 7)*

### The importance of integrating greening and nutrition education into the rest of the school program

Not only having the vision that greening the schoolyard and nutrition education are connected appeared to be important, also seeing the coherence between greening, nutrition education and the rest of the curriculum that schools have was mentioned by the interviewees as third important aspect influencing the feasibility and success of making the connection. Interviewees who see this coherence mentioned that by integrating greening and nutrition education well into the other education and activities the schools offer, the connection between the two can be made more easily. Moreover, according to these interviewees, integrating this connection can also provide a solution for hindrances such as lack of time and manpower because greening and nutrition education are not added on top of the existing lesson programs and activities but are woven into them.

*"Make it part of what you’re doing. Connect it to what you’re doing. So, if you work thematically, make sure you at least include a food theme. Then you don’t want: and, and, and. The experience in education is: all of that comes on top. This had to, this is imposed on it, and this is a must. And we need to get rid of that. We try to connect everything with everything. So, we gradually work thematically and have also said we want to connect nutrition education with the green yard. Hence the vegetable garden and a nutrition education curriculum. And completely experience it. It is truly completely interdisciplinary, geography, history, everything is in it. So, that is really, really beautiful*." *(Interviewee 8, school 7)*

To integrate greening the schoolyard, nutrition education, and the connection between them into the rest of the curriculum, interviewees mentioned that working thematically is very helpful. Most schools that already make the connection also work thematically, meaning that many areas of the curriculum are connected together and integrated within a theme. Additionally, according to interviewees, in this way it is also more accessible for teachers to get started with greening and nutrition education since they are not standalone activities but are integrated into the curriculum.

*"And don’t forget: everything you do is also language or math already.*" *(Interviewee 2, school 2)*

## Discussion

### Summary of the main results

This study aimed to understand the perspective of primary schools regarding the combination of greening schoolyards and nutrition education, and to assess the feasibility of this combination. The findings revealed that the schools in this study have undertaken greening and nutrition education initiatives in various ways over the years, with various factors, including vision, support and integration, influencing the feasibility to make a connection between the two.

Both this study and the research by Burt et al. [28] show that support among the school team is essential in making it feasible for schools to connect greening schoolyards and nutrition education. The study of Burt et al. investigated barriers in using school gardens in schools in the United States. Lack of support among the school team was found to be one of the main barriers in creating and using school gardens. Examples of this are lack of support in the garden maintenance or poor integration into the daily school curriculum due to a lack of interest of teachers [28], This confirms the results found in the current study. On the other hand, having support in the local community and having this community involved with greening the schoolyard was the most important determinant of a successful school garden, according to Burt et al. [28]. The role of the local community emerged also in the current study as important for the feasibility and successfulness of connecting greening schoolyards and nutrition education. However, the study of Burt et al. [28] suggests that involvement of the local community could have even more potential than found in this study. The study of Love et al. [29] confirms the latter and adds that an involved local community, for example by providing resources or supporting in the maintenance of the garden, is necessary for a sustained implementation of greening the schoolyard or nutrition education. Further research is needed to investigate the potential role that a local community can play, as well as the size of this role that is favorable, in making a connection between greening schoolyards and nutrition education.

Parental support also emerged as an important factor influencing the feasibility of making the connection between greening schoolyards and nutrition education in primary schools. Generally, according to the participants, parents were more supportive of greening initiatives than nutrition education, posing challenges to combining both. Literature suggests that greater parental support enhances the success of greening and nutrition education efforts, since family involvement and role modelling in such activities promote children’s engagement [30, 31]. In addition, Charlton et al. [30] recommend not only creating support among parents, but also integrating parental involvement into the activities. For example, allowing parents to participate in activities around greening or nutrition education and providing activities for pupils and parents at home [30]. Involving parents in activities related to greening or nutrition education was not explicitly investigated in the current study. However, the establishment of a green committee involving parents was found to increase support, which was also confirmed by the studies of Huys et al. [31] and Hoover et al. [32].

The origin of the vision of schools on the presence of the connection between greening the schoolyard and nutrition education could not be properly explained by the interviewees in this study. The study of Gonsalves et al. [33] found factors that can influence schools’ views on these themes, such as receiving advice from external parties or having the ability to think creatively. The results of that study also indicate that a lack of vision and ability to think creatively often results in a general negative perception of the themes of greening and nutrition education. This was not evident from the current study; schools that did not see the connection did not have a particularly negative perception of greening or nutrition education. Nevertheless, future research on how perspectives on connecting greening schoolyards and nutrition education develop can help other schools understand the connection, which can support more schools to make the connection themselves.

Integration emerged in this study as an important factor in seeing or not seeing the connection between greening and nutrition education. The interviewees that saw the connection also generally saw more coherence between different subjects and activities than interviewees that did not see the connection. Integrating greening or nutrition education into the general curriculum and into the culture of the school also appears in the literature as an aspect that makes working with greening and nutrition education feasible and more effective [34, 35]. According to Follong et al. [34], greening schoolyards and nutrition education can both be used as a context for many subjects such as mathematics, science or literacy courses, improving children’s vocabulary, crop knowledge and arithmetic skills, among other things. Van Dongen [25] emphasized that in the Netherlands, the Healthy School approach supports and stimulates schools to follow the principles of an integral approach. Remarkably, two of the three schools in this study that have a Healthy School-certificate have not implemented an integral approach and do not make the connection between greening the schoolyard and nutrition education. Future research can focus on the potential role of the Healthy School approach in the feasibility of greening the schoolyard and offering nutrition education.

In this study, no explicit questions were asked about why schools were involved in greening the schoolyard and/or in nutrition education. Follow-up research can focus on the reasons for schools to start working on these themes, which could be helpful for schools that have not yet started and want to do so.

### Strengths and limitations

A strength of this study includes the qualitative approach of this research. The use of qualitative research made it possible to gain insight into the vision on, and experience with combining greening schoolyards and nutrition education, because qualitatively research emphasizes patterns in behavior and experiences. Another strength lies in the sample size of this study. When executing qualitative research, the sample size is often too small to make the results generalizable. However, after conducting twelve interviews, data saturation occurred which increases the generalizability of the results.

However, for a correct interpretation of the results some methodological considerations should also be noted. The first limitation includes the absence of an ‘inter-coder agreement’ since the data has been coded by only one researcher. This increases the risk of subjectivity in interpreting the data. However, by thoroughly examining the data and discussing the pre-established codes, the categorization, and identified themes with colleagues involved in the research this risk was ought to be lowered. Secondly, the use of the theoretical framework by Proctor et al. led to two limitations in this study. The concept of penetration (the portion of the target audience using the intervention) from this framework was not included in this study because it was assumed that pupils would not have a choice in whether they would use a greened schoolyard or nutrition education. However, the results of this study revealed that pupils in some schools do have this choice because some schools offer elective courses in which pupils can enroll. Including this concept might have led to different results. Besides that, two concepts from the framework of Proctor et al., acceptability and appropriateness, appeared to be overlapping too much with each other during coding, making it difficult to differentiate them. Literature indicates that this is common when using this framework in qualitative research [17]. By closely following the description of the two concepts by Proctor et al., it was intended to successfully make a distinction between the concepts and ensure that the data was fully covered. Thirdly, the timing of this study could have been a limitation because this study was conducted just before the summer vacation which is often a hectic time for primary schools. Moreover, in the Netherlands, at the time of data collection, there was a significant staff shortage. This meant that school members were even busier than usual and had little availability for interviews. The timing and staff shortage-related pressures might have influenced the results. Future research could therefore take the timing of the execution of the data-collection into account.

## Conclusions

Combining greening the schoolyard and nutrition education in primary schools is perceived as feasible and can be successful when three factors are present: recognition of the connection by the school team and school directors; support from parents, the school team, pupils, and local community; and integration of greening the schoolyard and nutrition education into the existing school curriculum. Future research should investigate the vision of the local community and parents on combining greening the schoolyard and nutrition education, and the effects of combining greening schoolyard and nutrition education on pupils and their environment.

## Supporting information

S1 File Interview guide. (DOCX)

S2 File Code book. (XLSX)

S3 File QOREC checklist. (PDF)
