# Supplementary material for: The feasibility of combining greening schoolyards and nutrition education in primary schools: A qualitative study
Source: PLoS One. 2024 Nov 15;19(11):e0313773. doi: 10.1371/journal.pone.0313773 (PMC11567566; doi:10.1371/journal.pone.0313773)
Supplement: S1 File — (DOCX) [file pone.0313773.s001.docx]

**S1 File. Interview guide.**

| ***Introduction*** |  |
| --- | --- |
| Welcome | Thank you in advance for your time. |
| Introduction (if necessary) | My name is … and I work at the Louis Bolk Institute. I am currently working on the project 'Greening schoolyards and nutrition education' in which I will conduct interviews with primary school employees. |
| Introduce subjects | This interview will be about your experiences and vision on greening the schoolyard and offering nutrition education. |
| Aim of the research | I conduct these interviews to gain insight into how schools are working on greening schoolyards and offering nutrition education. Other schools can learn from this. |
| The interview | After this introduction, we will discuss four topics related to greening the schoolyard and offering nutrition education. After that, there is room for questions or comments. |
| Anonymity, right and wrong answers and voluntary participation | This interview is processed anonymously, which means that the results cannot be traced back to individuals (i.e. not traceable to what you said). There are no right or wrong answers. You may stop the interview at any time without consequences. Your participation is on voluntary basis. |
| Data usage | By law, the interview data must be kept for 10 years. This is kept in a secure environment and only people from the research team can access it. |
| Duration of the interview | The interview lasts half an hour to an hour. |
| Recording of the interview | I would like to record the interview so that I can transcribe it completely and analyze it later. When I start the recording in a moment, I will ask you if you want to give permission to record the conversation.  START RECORDING  Are you okay with this interview being recorded? |
| ***Start*** |  |
| Introduction of the interviewee | Would you like to introduce yourself?  O How long have you worked at this school?  O What is your position within this school? |
| **Subjects** |  |
| 1. Vision and experience | *First I want to talk about the role of the schoolyard and nutrition education at your school and how you fill this role in.*  Can you tell me what you have done with greening the schoolyard? (If a design is available, can I have it?)   - What is the area that has gone from tile to soil/green? If you don't know exactly, can you make an estimation? - Have you planted flowers, plants or trees? Created a vegetable garden? Or a play area for the pupils? Something else? - What did you think were the most important elements when greening the schoolyard; why did you want that?   Can you tell me how you have used the schoolyard this school year?   - For example, have the pupils played outside more or differently? Have more lessons been taught outside? - Have you used the vegetable garden (sowing, maintenance, harvesting, tasting, etc.)? - Have you paid attention to how food grows/nutrition education in a different way with the schoolyard?   Do you also pay attention (in a different way) to the theme of healthy and sustainable eating at your school, in addition to the vegetable garden, for example?   - If so, can you tell us what you have done this school year on the theme of healthy and sustainable eating? - Is it reflected in lessons or teaching methods/in activities, in themes/in policy (treat policy, drinking water, eating fruit and vegetables during the morning break)? - Do you as a school participate in Healthy School?   In addition to greening the schoolyard, do you also pay attention to the theme of nature and the environment in other ways at your school?   - Is this reflected in lessons or teaching methods, in activities, in themes or policy? - If so, how do you see that reflected in this?   What do you think about the fact that you are greening the schoolyard?   - Have you had a good experience with the greened schoolyard so far? - Have you also had less good experiences? - How relevant or useful do you find greening the schoolyard when it comes to the themes of healthy & sustainable food and nature & the environment? - Did the degree of relevance change over time?   (What do you think of the fact that you are working on the theme of healthy and sustainable eating/nutrition education in this way?   - Have you had a good experience so far with combining the green schoolyard and nutrition education? - Have you also had less good experiences? - How relevant or useful do you find providing nutrition education? - Did the degree of relevance change over time?)   Do you feel that greening the schoolyard and offering nutrition education has an influence on the pupils?   - If so, what does it influence, what added value does it have for the pupils, and in what way?   How important are themes such as healthy & sustainable food and nature & the environment for you? |
| 2. Decisions and implementation | *We have just talked about your experience with the greened schoolyard and nutrition education, now I want to talk about the process and implementation of greening the schoolyard and offering nutrition education.*  Are you involved in the school's decision to start greening the schoolyard?   - If yes,… - How do you as a school decide to start working on this? Is there one leader or how are the tasks divided? - How is support for this created among the school team and parents? - Can you manage that? Is everyone participating? If not, how do you deal with the fact that not everyone participates?   Are you involved in the choice of the school to offer nutrition education?   - If yes,… - How do you as a school decide to (not) work on nutrition education? - (How is support for this created among the school team and parents? - Can you manage that? Is everyone participating? If not, how will we deal with the fact that not everyone participates?)   Do you see a connection between greening the schoolyard and offering nutrition education?   - What does that connection look like? How is it related to each other? - Have you included the link in the design? Why/why not? - What would you need if you wanted to make that link (more)?   Are you able to use the greener schoolyard (and link nutrition education to it)?   - Did you expect that? - What did you hope for?   To what extent are you able to integrate the use of the green schoolyard (and any activities related to healthy and sustainable eating) into the other activities that you undertake as a school?   - Did greening the schoolyard and/or nutrition education conflict with other priorities within your work?   Are you satisfied with how your school has started with the greener schoolyard?   - What was your role in this?   (Are you satisfied with how your school has started offering nutrition education and the link to the greener schoolyard, e.g. through vegetable gardening?   - What was your role in this?) |
| 3. Needs and barriers | *This seems sufficient to me for now in terms of vision and experiences, I would now like to talk about the needs and barriers that you have experienced and are experiencing with the green schoolyard and the offering of nutrition education.*  What did you encounter when using the green schoolyard, providing nutrition education or combining these?   - What barriers have you encountered? - For example, budget, finding the right supporting parties, collaboration within the school team   What is your experience with finding and applying for subsidies? Were you able to find this and was it easy to apply?  Do you find it difficult to work on the greener schoolyard, providing nutrition education or combining these?   - If so… - How much effort does it cost you? - What would help you reduce this (if it is too much)?   Are you missing anything or do you have any wishes when getting started with the greener schoolyard?   - If so, what needs or wishes do you have/does the school have? - What else do you need for this?   Do you have any needs or wishes when offering nutrition education?   - If so, what needs or wishes do you have/does the school have? - What else do you need for this?   Do you have any needs or wishes when linking the green schoolyard to nutrition education?   - If so, what needs or wishes do you have/does the school have? - What else do you need for this? |
| 4. Successes and failures | *In addition to the needs and barriers you have experienced, I would also like to talk about the success and failure factors you have encountered when using the greened schoolyard and offering nutrition education.*  What do you think are the success factors or tips for getting started with a greener schoolyard?   - Does this differ depending on the activity you undertake on the greened schoolyard?   What success factors or tips can you think of when getting started with nutrition education?  What success factors or tips can you think of to make the link between the greener schoolyard and nutrition education?  In your opinion, what are the factors that other schools should take into account that could hinder the use of the greener schoolyard?   - Does this differ depending on the activity you undertake on the greened schoolyard? - How do you solve that?   What factors can you think of that could get in the way of getting started with nutrition education?   - How do you solve that?   And what factors could stand in the way of establishing a link between the greener schoolyard and nutrition education?   - How do you solve that?   Would you like to continue working on the greener schoolyard and nutrition education next year?   - Why/why not? |
| ***Ending*** |  |
| End of the interview | This was my last question. Are there any topics that we have not discussed, but that you think are important to mention?  What did you think of the interview? |
| Future research | This interview will be fully transcribed and compared with other interviews. The results are presented in a Dutch-language report as well as a scientific article. You will receive the results of the study when they are available. |
| Questions or comments | Do you have any questions or comments about the interview? |
| Thanking | Thank you for your time and cooperation! |
